# FR database 1.0: a resource focused on fruit development and ripening

**DOI:** 10.1093/database/bav002

**Published:** 2015-02-27

**Authors:** Junyang Yue, Xiaojing Ma, Rongjun Ban, Qianli Huang, Wenjie Wang, Jia Liu, Yongsheng Liu

**Affiliations:** ^1^School of Biotechnology and Food Engineering, Hefei University of Technology, Hefei 230009, China, ^2^School of Medical Engineering, Hefei University of Technology, Hefei 230009, China, ^3^School of Information Science and Technology, University of Science and Technology of China, Hefei 230009, China, ^4^Ministry of Education Key Laboratory for Bio-resource and Eco-environment, College of Life Science and ^5^State Key Laboratory of Hydraulics and Mountain River Engineering, Sichuan University, Chengdu 610064, China

## Abstract

Fruits form unique growing period in the life cycle of higher plants. They provide essential nutrients and have beneficial effects on human health. Characterizing the genes involved in fruit development and ripening is fundamental to understanding the biological process and improving horticultural crops. Although, numerous genes that have been characterized are participated in regulating fruit development and ripening at different stages, no dedicated bioinformatic resource for fruit development and ripening is available. In this study, we have developed such a database, FR database 1.0, using manual curation from 38 423 articles published before 1 April 2014, and integrating protein interactomes and several transcriptome datasets. It provides detailed information for 904 genes derived from 53 organisms reported to participate in fleshy fruit development and ripening. Genes from climacteric and non-climacteric fruits are also annotated, with several interesting Gene Ontology (GO) terms being enriched for these two gene sets and seven ethylene-related GO terms found only in the climacteric fruit group. Furthermore, protein–protein interaction analysis by integrating information from FR database presents the possible function network that affects fleshy fruit size formation. Collectively, FR database will be a valuable platform for comprehensive understanding and future experiments in fruit biology.

**Database URL**: http://www.fruitech.org/

## Introduction

Fruits are derived from flower gynoecium, which is only strictly found in the angiosperms. They are normally defined as structures originated from the fertilization of ovules, including dry fruits (e.g. the model plant: *Arabidopsis*) and fleshy fruits (e.g. the model for fleshy fruits: tomato). Studies on *Arabidopsis* and tomato have showed significant similarities between dry and fleshy fruits in the molecular circuits governing development and ripening (1). Dry fruits and fleshy fruits share a well-conserved, protracted and complex process in most basic physiology which involves many genes and common activities, especially at stages of fertilization and early development of fruit. In both dry fruits and fleshy fruits, fruit growth is stimulated by gibberellins (GA) signal whose biosynthesis is upregulated upon the generation of a seed-originating auxin signal (2). Consistent with this, reduction on the activity of the growth-repressing DELLA proteins, which play a central role in GA signaling, can promote parthenocarpic fruit development (2, 3).

Dry fruits become lignifications during extended periods, while fleshy fruits will turn out juicy and sweet. These attractive properties of fleshy fruits are not only ready for consumption in the raw state, but also with respect to the health-promoting compounds of particular fruit traits. The importance of fleshy fruits in human diet encourages researchers to find solutions on the following questions which are not well concerned for dry fruits: (i) What are the mechanisms that regulate the size and shape of fleshy fruits during developmental stages characterized by cell division and expansion? (ii) What leads to the marked changes in texture, color and flavor during the ripening of fleshy fruit? and (iii) How to control the rate of softening and extend shelf life of fleshy fruits during post harvest (PH) period?

To encompass these processes progressively, the growth duration of the majority of fruits (especially the climacteric fruits) has been typically divided into the following distinct stages characterized by cell division and expansion, color transition and pigments accumulation as well as fruit softening: immature fruit (IF), mature green (MG), breaker (BR), ripe fruit (RF) and PH (4). At the IF stage, developing seeds inside the ovary produce hormones (e.g. cytokinins and gibberellic acid) which promote cell division and expansion. Once ovary size reaches the maximum, another hormone named abscisic acid will be produced and lead to seed maturation and dormancy. In fruits that lack seeds, treatments with exogenous dilute solution of gibberellic acid is able to promote growing to their larger fruit size. At the MG stage, fruits reach full size, but still stay hard, green and sour without fragrant smell. Some enzymes (e.g. hydrolase, amylase and pectinase) start to express and catalyze hydrolyzation of starch into sugar, digestion of pectin and some other processes. BR stage is a transition period exhibiting the color change of fruit skin caused by chlorophyll degradation. This stage marks the onset of fruit ripening. During ripening stage, chlorophyll is broken down and new pigments that make fruit color red, yellow or blue are produced. Fruit ripening is a genetically regulated process which involves the accumulation of aroma and flavor, softening of fruit tissues as well as increased susceptibility to opportunistic pathogens. Based on the presence or absence of enhanced respiration and synthesis of the gaseous hormone ethylene at the onset of ripening, fruit species are classified as climacteric fruit and non-climacteric fruit (5). In PH stage, fruits are detached from their mother plants, but the respiration and softening processes are still ‘running’ and influenced by particular genes. For example, shelf life is extended by modulation on genes encoding α-mannosidase and β-d-*N*-acetylhexosaminidase, which could slow down fruit softening rates (6). In addition, some fruits manifest unique species-specific characteristics during development and ripening, such as the white stage in strawberry (7). For instance, at white stage, the contents of flavonols and anthocyanins as well as the expression levels of their pathway genes are shown to be decreased in comparison with green stage, but increased during the turning stage (8).

Although, lots of genes/proteins have been found to be involved in cellular processes, stress resistance, pigmentation and nutrient accumulation during fruit development and ripening, no integrated web-accessible platform which is unique to the fleshy fruit is available. For intensive studies in this area, we have developed a searchable database, FR database 1.0, and describe it here. In contrast to species specific databases (9–11), our database contains as many fleshy fruits as possible to facilitate further investigations across species. It contains comprehensive information for 904 genes that have been reported to participate in fruit development and ripening by manual curation from 38 423 articles covering 53 organisms. Users can find genes of interest by searching our web-server-based FR database 1.0. It will provide detailed information for the query genes/proteins including: (i) the sequence and basic information; (ii) the protein and structural annotation and (iii) the expression and biological function. Furthermore, FR database 1.0 provides two additional advanced options for users. FR database 1.0 is implemented in PHP +  MySQL +  JavaScript and can be accessed at http://www.fruitech.org without registration.

## Methods

**Figure 1.**
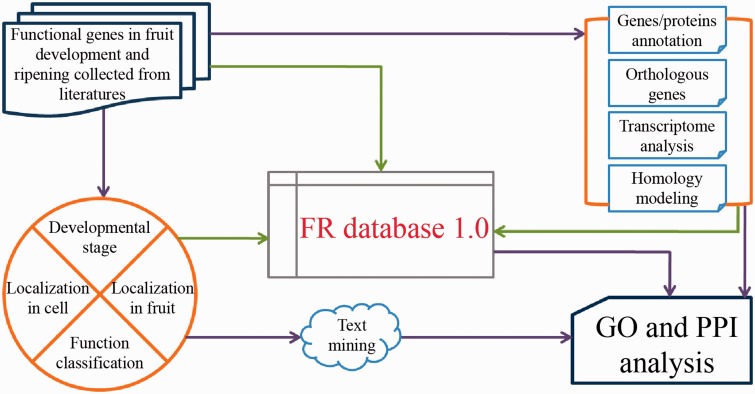
FR database 1.0 scheme.

The general process of data collection, annotation and model development is illustrated in Fig. 1.

### Manual curation of literature

With the aim of creating a curated FR database with high quality, we searched PubMed with the keywords of ‘fruit’ and ‘fruits’ to include all literatures that relate to fruit development and ripening. After subsequent data processing, such as removing the duplicates and deleting the topics related to ‘fruit fly’, we kept 38 423 articles published before 1 April 2014 for collecting genes/proteins identified to be functional in fruit development and ripening. Only those genes with experimentally verified functions occurring in fruit development and/or ripening were collected in our database. In total, we collected 904 unique genes (here called ‘functionally known genes in fruit development and ripening’) from 53 organisms, in which the 5 most extensively studied fruit species are *Solanum lycopersicum* (29.31%), *Malus domestica* (8.19%), *Vitis vinifera* (7.85%), *Fragaria ananassa* (6.86%) and *Musa acuminata* (5.75%), respectively. The distributions of these functional genes characterized by developmental stages, subcellular localization and tissue-specific expression during fruit development and ripening are listed in Supplementary Tables S1, S2 and S3.

### Annotation of each gene

After collecting all the experimentally verified genes, we annotated them as follows: (i) basic information [e.g. name/synonyms, nucleotide sequences, mutations (12), protein sequences, CDS site, theoretical PI and molecular weight (MW)] of the genes/proteins is annotated referring to UniProt Knowledgebase and GenBank; (ii) protein structure, functional domain [covering the annotations from PIRSF (13), Pfam (14), SUPFAM (15) and Prosite (16) database] and the protein–protein interaction (PPI) information [combination of the records from BioGRID (17), DIP (18), MINT (19), IntAct (20) and String (21) database] are provided and (iii) biological function and expression information including GO annotation and detailed description from experimental data (e.g. developmental stages, subcellular localization, differential fruit tissues, analysis method and function classification) are also provided together with the corresponding figures and titles of literatures.

FR database 1.0 is constructed as an integrated bioinformatic resource and implemented in PHP + MySQL +  JavaScript. Its online documentation contains the help information (USAGE) and the frequently asked questions to guide new users to use this resource (http://www.fruitech.org/documentation.html).

### Integration of transcriptome data

The transcriptome datasets of our target organisms were downloaded from the gene expression omnibus repository and sequence read archive database as well as some other open sources (Supplementary Table S4). All the data related to genes harbored in our database was extracted. Then, the heatmap figures were made based on relative RPKM value. Corresponding data could be easily viewed from our database. In addition, the search function provides access to detailed descriptions of individual genes, functional information and possible FR ID.

### Establishment of PPI

Based on the interactomes established with experimental verifications of Arabidopsis (*Arabidopsis thaliana*) (22), nematode worm (*Caenorhabditis elegans*) (23), fruit fly (*Drosophila melanogaster*) (24), human (*Homo sapiens*) (25) and yeast (*Saccharomyces cerevisiae*) (26), we predicted the potential PPIs among genes/proteins involved in our database through comparing the orthologous genes. Thus, 1246 non-redundant PPIs, consisting of 405 intra-specific and 841 inter-specific PPIs, were obtained (Supplementary Table S5). Then, the Cytoscape Web was embedded within our web pages to display these identified PPI datasets (27).

### Construct of PDB structure

The PDB structures were first obtained from RCSB–PDB database where their crystal structures have been solved by the experimental methods. Alternatively, the PDB structures were built based on their amino acid sequences through homology modeling using the fully automated server, SWISS-MODEL, which referred to target proteins with known experimental three-dimensional structures (28). Then, the PDB structures were visualized using Jmol, a web browser applet integrated into our web pages (29).

## Utility

### Simple search

**Figure 2.**
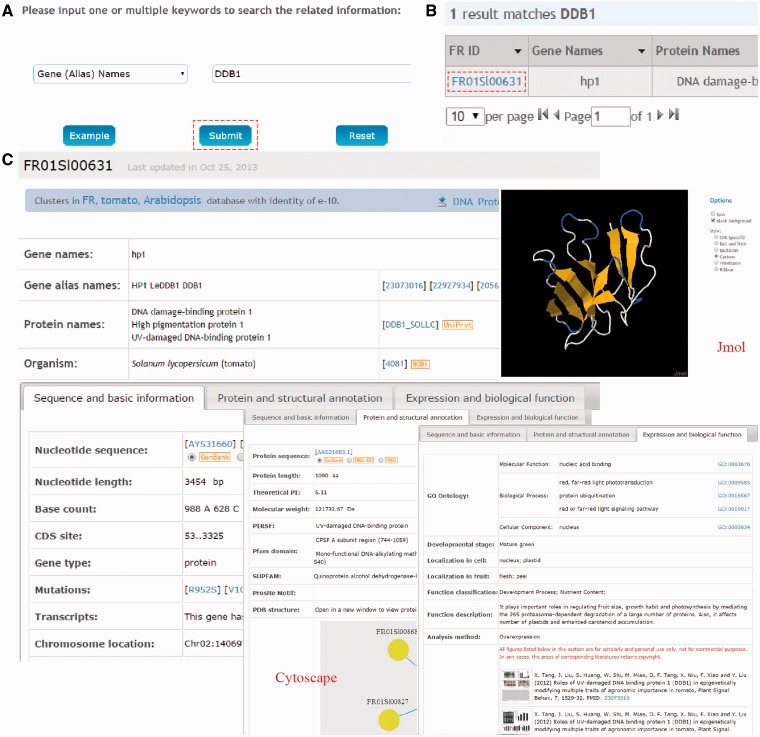
The search function of FR database 1.0. (**A**) Users can simply input gene/protein ‘DDB1’ for querying. (**B**) The results are shown in a tabular format. Users can visualize the detailed information by clicking on the FR ID (FR01Sl00631). (**C**) The detailed information for tomato DDB1 (hp1). The information presented here has been checked and will be updated based on new data published.

FR database 1.0 is developed in an easy-to-use mode, providing a search engine for users to find the genes of interests. The search option (http://www.fruitech.org/search.html) provides an interface for querying the FR database 1.0 with one or several keywords (gene/protein names) or accession numbers (UniProt ID or FR ID). For example, if a keyword of DDB1 were input and submitted (Fig. 2A), the search results will be shown in a tabular format, containing FR ID, Gene Names, Protein Names, UniProt ID, Species and Developmental Stage (Fig. 2B). By clicking on the FR ID (FR01Sl00631), the detailed information for tomato gene/protein *hp1* (DDB1) will be shown (Fig. 2C) as three parts: (i) sequence and basic information (e.g. Gene names, Nucleotide sequence, Base count and Number of transcripts); (ii) protein and structural annotation [Protein sequence, Theoretical PI, MW, PDB structure, PPIs and other database annotation (PIRSF, Pfam domain, SUPFAM and Prosite Motif)] and (iii) expression and biological function (GO annotation, Function in developmental stages, Cell and fruit localization, Function classification, Figures and Literature information).

### Advanced search

Furthermore, FR database 1.0 provides two additional advanced options: (i) Advanced search: in this option, users can use up to three search terms with relatively complex or combined keywords to locate the precise information. The interface of search engine permits querying by combination of different annotation fields using ‘and’, ‘or’ or ‘exclude’. (ii) Homologous search: to search homology for special gene in several species, users can browse homologous information by providing the FR ID of a gene/protein. For example, by clicking on the ‘Example’ and ‘Submit’ button successively, the homologous information (identity ≥ 60%, *E*-value ≤ e−10 and score ≥ 100) of DDB1 (FR ID: FR01Sl00631) in *A.* *thaliana* will be shown, with gene names and other detailed results from the blast program (30).

## Results and Discussion

Fleshy fruit development and ripening is a multi-faceted but tightly regulated process responsible for fruit enlargement, texture alteration and nutrients accumulation. Many critical molecules involved in this process have been identified. On fertilization, the precursor of the fleshy fruit enters a stage at which cell division and expansion are immediately followed, but without lignifications which are developed in dry fruit. Due to large part to the use of tomato models, lots of data have been obtained ranging from IF to PH stage. Combined with data from other species, it is demonstrated that many genes functioning in tomato also play conserved roles across a wide spectrum of fleshy fruit species and *vice versa*, mainly because of evolutionary and functional conservation among diverse species (31–33). To effectively analyse the data generated in various experiments using different species, it is necessary to collect and manage these data in an organized manner.

**Figure 3.**
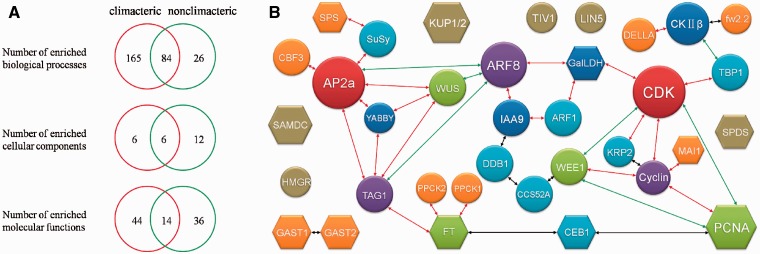
(**A**) GO analysis for genes in climacteric and nonclimacteric fruit. (**B**) The potential protein network affecting fruit size during development. Circle stands for proteins in tomato. Hexagon represents proteins from other fleshy fruit except tomato. Different color indicates different number of interactions. Size of shape has no meaning. The red line indicates an interaction with middle confidence. The green line means an interaction with lower confidence. The black line indicates an interaction from text mining analysis.

Unlike other existed reproductive-related database in plant: (i) SeedGenes (34) only concentrates on *Arabidopsis*; (ii) RAPESEED (35) emphasizes on seed development for oilseed crops, FR database 1.0 focuses on the process of fleshy fruit development and ripening including 904 experimentally verified genes derived from 53 organisms. By carefully inspecting the literatures, fleshy-fruit associated genes have been collected, annotated and classified. Since fleshy fruits have historically been divided into climacteric (e.g. tomato, banana, apple) and non-climacteric (e.g. grape, strawberry, citrus) based on whether they manifest a burst of respiration at the onset of ripening. We collected and submitted those genes from both climacteric and non-climacteric fruit groups to GO enrichment analysis separately. We statistically calculated the represented biological processes, molecular functions and cellular components for the distribution of all genes (Hypergeometric distribution, *P*-value < 0.05, enrichment fold > 2; Details of GO results are presented in Supplementary Table S6). Some GO terms were enriched for genes from both climacteric and non-climacteric fruit groups, mainly concentrating on cell growth and morphogenesis, metabolic process as well as stress response. Interestingly, seven GO terms related to ethylene process were only enriched for genes from climacteric fruits; rather, none of these GO terms was enriched for genes from non-climacteric fruits. For example, response to ethylene stimulus (GO: 0009723) was enriched by 35 counts with climacteric fruits. In the GO-represented biological processes, 84 GO terms were enriched for these two sets of fleshy fruits genes, 6 GO terms were enriched in GO-represented cellular components and 14 GO terms in GO-represented molecular functions (Fig. 3A). This analysis was in accordance with the classification principles, further indicating that FR database could facilitate understanding of regulatory mechanisms during fruit development and ripening.

Fruit development and ripening must be, at any time, dictated by networks of genes expressed. Thus, PPI information of these genes/proteins would be critical for understanding the mechanisms of these processes. Using experimentally validated PPIs (PPI records from BioGRID, DIP, MINT, IntAct and String database) as background, we constructed a primary protein network including the genes with effect on fruit size at different developmental stages (Fig. 3B). All these genes are actually involved in cell division and expansion. For example, *fw2.2* has been shown to be responsible for approximately 30% enlargement of final size of tomato by negatively regulating mitosis (36), while LeKRP1 and LeKRP2 are demonstrated to affect fruit size mainly through endoreduplication without cell division (37). Obviously, these proteins are involved in cell cycle signal transduction pathway and predicted to interact with protein CDK directly or indirectly. In contrast, DDB1 alters fruit size via an epigenetic manner and mediates the 26S proteasome-dependent degradation of proteins (such as epigenetic marks and cell cycle-related proteins) (37, 38). Many transcription factors, such as MADS (39), ARF (40), bHLH (41) and TCP (42), also play a critical role in cell cycle regulation. In addition, *fasciated* (43) and WUSCHEL (44) control fruit size by determining carpel number rather than cell cycle. All of the above-mentioned proteins would impose an effect on final size of fruit formation. Our analyses have showed the potential interactions that contain all the identified fruit-size-associated proteins in FR database 1.0. For example, our database predicts that cell cycle and cell number are likely regulated by ARFs (transcription factors; Fig. 3B). But how these proteins work and interact with each other warrant further in-depth study. Moreover, FR database 1.0 also provides some other potentially interesting interactions whose functions in fruit development and ripening are unknown.

Taken together, we have developed a resource named FR which presents a comprehensive platform for users to access the collective information of experimentally verified genes involved in fruit development and ripening. It integrates the detailed information of functional characterization for 904 genes. FR database 1.0 will help researchers to obtain a comprehensive resource for understanding the complex biological mechanisms of fruit development and ripening.

## Funding

Supported by the National Science and Technology Key Project of China [2011CB100401]; the National Science Fund for Distinguished Young Scholars [30825030]; the National Natural Science Foundation of China [31171179, 31471157 and 31461143008]; Advanced Program of Doctoral Fund of Ministry of Education of China [20110181130009]; a Key Project from the Government of Sichuan Province [2013NZ0014]; a Project from the Government of Anhui Province [2012AKKG0739]. Funding for open access charge: the National Natural Science Foundation of China [31461143008].

## Supplementary Data

Supplementary data are available at *Database* Online.

*Conflict of interest*. None declared.
